# *Bacillus amyloliquefaciens* Enriched Camel Milk Attenuated Colitis Symptoms in Mice Model

**DOI:** 10.3390/nu14091967

**Published:** 2022-05-08

**Authors:** Ashraf Khalifa, Abdullah Sheikh, Hairul Islam Mohamed Ibrahim

**Affiliations:** 1 Biological Science Department, College of Science, King Faisal University, P.O. Box 400, Al-Ahsa 31982, Saudi Arabia; akhalifa@kfu.edu.sa; 2 Botany and Microbiology Department, Faculty of Science, Beni-Suef University, Beni-Suef 62511, Egypt; 3 Camel Research Center, King Faisal University, P.O. Box 400, Al-Ahsa 31982, Saudi Arabia; asheikh@kfu.edu.sa; 4 Molecular Biology Division, Pondicherry Centre for Biological Sciences and Educational Trust, Kottakuppam 605104, India

**Keywords:** inflammatory bowel disease, probiotics, *Bacillus amyloliquefaciens*, TNF-α, PCNA

## Abstract

Fermented camel’s milk has various health beneficial prebiotics and probiotics. This study aimed to evaluate the preventive efficacy of *Bacillus amyloliquefaciens* enriched camel milk (BEY) in 2-, 4- and 6-Trinitrobenzenesulfonic acid (TNBS)-induced colitis mice models. To this end, the immune modulatory effects of *Bacillus amyloliquefaciens* (BA) on TNF-α challenged HT29 colon cells were estimated using the cell proliferation and cytokines ELISA method. BEY was prepared using the incubation method and nutritional value was quantified by comparing it to commercial yogurt. Furthermore, TNBS-induced colitis was established and the level of disease index, pathological scores, and inflammatory markers of BEY-treated mice using macroscopic and microscopic examinations, qPCR and immunoblot were investigated. The results demonstrate that BA is non-toxic to HT29 colon cells and balanced the inflammatory cytokines. BEY reduced the colitis disease index, and improved the body weight and colon length of the TNBS-induced mice. Additionally, Myeloperoxidase (MPO) and pro-inflammatory cytokines (IL1β, IL6, IL8 and TNF-α) were attenuated by BEY treatment. Moreover, the inflammatory progress mRNA and protein markers nuclear factor kappa B (NFκB), phosphatase and tensin homolog (PTEN), proliferating cell nuclear antigen (PCNA), cyclooxygenase-2 (COX-2) and occludin were significantly down-regulated by BEY treatment. Interestingly, significant suppression of PCNA was observed in colonic tissues using the immunohistochemical examination. Treatment with BEY increased the epigenetic (microRNA217) interactions with PCNA. In conclusion, the BEY clearly alleviated the colitis symptoms and in the future could be used to formulate a probiotic-based diet for the host gut health and control the inflammatory bowel syndrome in mammals.

## 1. Introduction

Inflammatory bowel disease (IBD) is a group of diseases including ulcerative colitis that is noted by chronic inflammation in the gut causing bloody diarrhea, abdominal pain and weight loss [1]. The causes of the disease are most likely immune disorders and unhealthy food habits. The European Crohn’s and Colitis Organization recommended detailed guidelines for therapeutics [2]. The incidence of IBD is increasing in the Arab world [3]. Searching for a natural and safe fermented food source is a promising approach to treating IBD disease.

Functional fermented foods from animal sources receive much interest nowadays, as they contain several health beneficial prebiotics and probiotics. Camel milk has a unique chemical composition and possesses well-documented therapeutic activities in treating many diseases, including autoimmune disorders such as IBD. It has recently been demonstrated by He et al. [4] that camel milk modulated the gut microbiota and had anti-inflammatory effects in a mouse model of colitis via maintaining intestinal barrier function and inhibiting proinflammatory cytokines in colon tissue. The gut microbiota is in a dynamic interaction with human hosts in various mechanisms, including modulation of epithelial functions, immune responses, and protection against microbial pathogens. The imbalance in gut microbiota is a condition known as dysbiosis, which causes various health problems, e.g., IBD.

Probiotics are live microorganisms that have multiple host health benefits. Many probiotic bacterial species from different sources have been reported to control IBD, including *Lactobacillus casei* from prato cheese [1], *Propionibacterium freudenreichii* from Emmental cheese [5], *Lactobacillus rhamnosus* from goat milk [6], *Bacillus subtilis* from neonate feces [7] and BA from traditional Korean fermented foods [8].

BA is a common probiotic bacterium that ameliorated colon inflammation in a dextran sulfate sodium (DSS)-induced IBD mice model [9] via lowering the levels of inflammatory cytokines (e.g., IL1β, IL4, IL6, IL8 and TNF-α) in colon tissues [8,10]. However, the beneficial role of BA in another pathological model, trinitrobenzenesulfonic acid (TNBS)-induced colitis, is still not fully understood. Therefore, the current study aimed to investigate the preventive efficacy of BA-supplemented camel milk in treating ulcerative colitis on a murine inflamed colon using a TNBS-induced colitis model. The levels of pro-inflammatory markers and the anti-inflammatory cytokine TGF-β in the distal colon were estimated in the treated and control mice groups. Furthermore, the inflammatory mRNA and protein markers were determined using quantitative PCR and immunoblot techniques. A histopathological analysis of the colon was also conducted for delivering information on alleviation levels of the inflammatory colon. The role of epigenetic short RNA in the TNBS-induced colitis model was quantified.

## 2. Materials and Methods

### 2.1. Bacterial Strain and Culture Conditions

BA (JF836079) was previously isolated and characterized for its probiotic traits [10]. Further characterization of probiotic traits, immunomodulatory and anti-oxidative features were carried out in a TNBS-induced colitis model. BA was preserved at −80 °C in De Man, Rogosa and Sharpe agar (MRS) cryostock and was sub-cultured in MRS broth for 18 h at 37 °C before use. Checking the identity of the strain was done by using Gram staining microscopic examination.

### 2.2. Effect of Probiotics on Viability of HT-29 Colon Cells Using Trypan Blue Assay

The cytotoxicity of BA against the human epithelial HT-29 cell lines was carried out as mentioned earlier by Hairul et al. [11]. Briefly, at 80% confluent, HT29 cell lines were trypsinized and the cell line was incubated with BA, at 10^8^ CFU/mL of PBS at 37 °C for 2 h. For checking the cell’s viability, BA-treated cells were aspirated with a fresh tissue culture medium with penicillin-streptomycin (100X) and incubated for 20 min. Then, they were aspirated again with a fresh culture medium without antibiotics to remove dead BA and cell debris. Monolayer HT29 cells were trypsinized and exactly 100 μL of cell suspension was added to 100 μL of 0.2% Trypan blue dye (0.5% in PBS), mixed gently, and then incubated at 25 °C for 3 min. Total numbers of the dead and live cells were estimated using a Biorad cell counter (Bio-Rad, Hercules, CA, USA).

### 2.3. Immunomodulation of TNF-α-Induced HT-29 Cells by BA

Immunomodulation of TNF-α-induced HT-29 cells by BA was determined using the cytokine estimation method [12]. Briefly, HT-29 cells (5 × 10^5^ cells/well) were grown in 24-well plate until confluence. In co-culture experiments, BA was added at concentrations of 10^8^ CFU/mL. Incubation of cells was carried out in the presence of BA with or without TNF-α (5 ng/mL) (PeproTech, London, UK), for 6 h. Then, cell-culture supernatants were stored at −80 °C until use. The concentrations of interleukine-1, IL-4, IL-6 and IL-8 in supernatants were determined using a GENWAY mammalian ELISA (GENWAY, CA, USA) following the recommendations of the manufacturer. The experiments were carried out in triplicate.

### 2.4. Preparation of Yogurt

Camel milk was pasteurized at 68 °C for 20 min, cooled in a cold water bath to 42 °C, and gently mixed and inoculated with BA under aseptic conditions [13]. Yogurt preparations were divided into two groups: (a) Natural yogurt (control without BA) and (b) BA-enriched Yogurt (inoculated with ≥10^8^ CFU/g of the BA). The produced yogurt was aseptically poured in sterilized polystyrene cups and incubated at 40 °C for ~5 h, until the pH reached 4.5  ±  0.05. Then, the yogurt was immediately cooled and stored at 4 °C (24 h), for cold maturation as well as further use.

### 2.5. Animal Care and Experimental Design for TNBS-Induced Colitis

Experiments were conducted on six-week old male C57Bl6j mice whose body weight ranged from 18 to 22 g that were obtained from the Animal House, College of Science, King Faisal University (KFU). All experiments on animals were conducted in full compliance with the code of ethics for the care and use of laboratory animals of the deanship of scientific research ethical committee (KFU-REC-2021-OCT-EA00075). Feeding of control group mice (non-colitis) was carried out by providing 5 g of a normal basal diet per animal per day and drinking water was supplied ad libitum. Mice were randomly divided into five groups (six animals per group). Two groups (non-colitic and control groups) received no probiotic treatment and the third and the fourth groups (treated group) were given the probiotics orally (5 × 10^8^ CFU suspended in 0.5 mL of skimmed milk (vehicle)) daily for 3 weeks. The third group continued for TNBS induction and the fourth group for probiotics alone. The non-colitic and control groups received probiotics orally (PBS as a vehicle of administration) (0.5 mL daily). The fifth group was considered as a TNBS alone group. Briefly, two weeks after starting the experiment, the mice were fasted overnight for intracolonical TNBS induction and were rendered the colitic group [14]. Briefly, they were anesthetized with isoflurane and given 50 mg/kg (BW of a mouse) TNBS dissolved in 0.25 mL of 5% ethanol by means of a Teflon cannula inserted 4 cm through the anus. Mice from the non-colitic group were intracolonically administered 0.25 mL of PBS instead of TNBS.

### 2.6. TNBS-Induced Colitis

TNBS (50 mg/kg BW) was instilled by a suitable medical-grade catheter for approximately 4–6 cm proximal to the anal verge (Carbosynth, China) and BA constituted yogurt during the entire duration of the experiment (28 days); however, colitis was induced similarly to the diseased control. Simultaneously, the animals of both experimental groups were also challenged with TNBS after the 22nd day. On the 36th day (2 weeks of TNBS induction), tissue samples were obtained from euthanized mice for assessment of the various traits. Euthanization was carried out by treating mice with an overdose of diethyl ether. Clinical signs such as hemoccult, diarrhea and body weight were observed daily for mice over the colitis period.

### 2.7. Assessment of Disease Activity Index (DAI)

The DAI was determined by summation of the clinical score reported on a daily basis for 6 days after colitis induction considering stool consistency (0: normal; 2: loose stool; 4: watery diarrhea), bloody stools (rectal bleeding) (0: normal; 2: slight bleeding; 4: gross bleeding) and weight loss (0: none; 1: decreased 1–5%; 2: decreased 5–10%; 3: decreased 11–15%; 4: decreased > 15%).

### 2.8. Bacterial Count in the Fecal Sample

In order to determine bacterial counts, mice feces were collected in sterile tubes before sacrificing animals on the 29th day and were homogenized in sterile PBS (pH 7.4). Serial dilution of homogenates was performed using the standard method. One milliliter from the 1:10^5^ dilution was plated on MRS and EMB agar. Bacterial counts were determined after 24–48 h of incubation at 37 °C, using a colony counter.

### 2.9. Tissue Collection and Sample Preparation

For hematological analysis, blood samples were collected by cardiac puncture. In the histologic examination, the intestinal fluid was collected after flushing the small intestine of euthanized mice with 2.5 mL of PBS (pH 7.4), followed by teasing with sterile needles in the same medium to separate the cells. Then, supernatant free of debris was obtained by centrifugation at 2000× *g* for 30 min at 4 °C. Next, the colonic tissues were cut and fixed in formalin, for staining with eosin (H&E) and hematoxylin and immune-histochemistry studies. Parts of intestinal samples were kept at −20 °C, for the subsequent RT-qPCR work.

### 2.10. In Vivo Permeability Assay

Intestinal barrier function was investigated in TNBS-induced mice (22 days after the second injection), using an in vivo permeability assay. Mice were administrated intragastrically with fluorescein-conjugated fluorescein isothiocyanate (FITC)-labeled dextran, a permeability tracer (0.6 mg/g of body weight, molecular weight 3000–5000 Da, Sigma-Aldrich, St. Louis, MO, USA) [15]. After 3.5 h, blood samples were obtained from the retro-orbital venous plexus, and FITC-dextran was estimated in the serum with a Multimode Plate Reader (Molecule device, fluorometer) at an emission wavelength of 535 nm and an excitation wavelength of 485 nm. Determination of FITC-dextran was estimated from the standard curve of FITC-dextran (5–400 ng) in non-treated serum (diluted 1:10 *v/v* with PBS).

### 2.11. Myeloperoxidase (MPO) Activity

The activity of MPO, a marker of neutrophilic infiltration, was determined in the distal colon, according to the method described earlier [16]. Briefly, the colon (~1 cm length) was obtained and homogenized (50 mg/mL) in ice-cold 50 mM PBS (pH 6) containing 5% hexadecyl trimethyl ammonium bromide (Sigma-Aldrich). After centrifugation, exactly 50 μL of the supernatant was mixed with o-dianisidine and hydrogen peroxide (H_2_O_2_) (Sigma-Aldrich). The absorbance was measured for the colorimetric reaction using a spectrophotometer (Tecan, Männedorf, Switzerland). MPO activity is expressed as units per milligram of wet tissue. One unit expresses the MPO activity needed for the conversion of 1 mM of H_2_O_2_ to water in 1 min at room temperature.

### 2.12. Markers of Inflammation in the Intestinal Tissue

The quantities of cytokines (TNF-α/IL-1β, IL-4/) were determined in the intestinal fluid using ELISA kits (Invitrogen, Thermo Fisher Scientific, Vienna, Austria; eBioscience San Diego, CA, US and Biolegend Inc., San Diego, CA, USA) following the instructions of the manufacturers. The levels of cytokines in the plates were reported at 450 nm on an automated ELISA plate reader (BioTek Instruments, Winooski, VT, USA). The C-reactive protein (CRP) in the intestinal fluid was also estimated using pre-coated anti-CRP (Boster Immunoleader, Pleasanton, CA, USA) microplate strips. Total CRP was determined at 450 nm of absorbance using a microplate reader (BioTek Instruments). CRP was expressed in mg/dL.

### 2.13. Transcriptional Expression of Inflammatory Genes

Transcriptional expression of inflammatory genes was carried out to investigate the effect of BEY on mice mRNA expression of immune genes using RT-qPCR. Extraction of total RNA from mice colonic tissues was carried out using Trizol Reagent (Sigma-Aldrich, St. Louis, MO, USA) following the manufacturer’s protocol. Purified RNA was quantified by measuring the absorbance at 260/280 nm using a micro-volume spectrophotometer (BioTek Instruments). Complementary DNA (cDNA) was generated from RNA (1 μg) using the rapid cDNA kit (Thermo scientific, MA, USA) according to the instructions of the manufacturer. Forward and reverse primers targeting the COX-2, NFΚB, PCNA, PTEN and occludin (Table 1) were used. The U6 and miR-217 primer sequences are in Table 1. The RT-qPCR was carried out to study expression levels of the target genes using the VII7A applied biosystemsthermocycler system (Applied Biosystems, Foster City, CA, USA). GAPDH served as an internal control. The qPCR (10 μL) was performed by using 1 μL of diluted cDNA (5 ng), 5 μL of SYBER green (Thermo Fisher Scientific), 0.5 μL of each primer (0.5 µM) and 3 μL of nuclease free water. The fluorescence signal was collected at the end of the reaction at 72 °C, and the lysis curve was set up from 65 to 95 °C with 0.5 °C/5 s increments. The results were evaluated based on the exponential growth of the cyber green signal, quantification cycle (Cq) values and dissolution curves. The data were analyzed using the 2^−ΔΔCt^ method, where the GAPDH was used as an endogenous control gene. The changes in transcript level fold were then determined in the treated and control groups, from threshold cycle (Ct) values according to the 2^ΔΔCt^ Livak and Schmittgen method [17].

### 2.14. Western Blot

The protein lysate was prepared from the distal colons of the TNBS- and BEY-treated mice groups using RIPA lysis buffer (Santa Cruz, CA, USA). The lysates was then separated on an SDS-PAGE gel (10%) and transferred to a PVDF (0.22 μm) membrane. Western blot assays were performed with the aid of specified primary antibodies: NFKB (mouse monoclonal antibody 1:1500) (Invitrogen, Waltham, MA, USA), PTEN (rabbit polyclonal antibody 1:1000) (Invitrogen, Waltham, MA, USA), PCNA (rabbit polyclonal antibody 1:1000) (Biorbyt, Cambridge, UK), COX2 (rabbit polyclonal antibody 1:1000) (Biorbyt, Cambridge, UK), occludin (mouse monoclonal antibody 1:1500) (Invitrogen, Waltham, MA, USA) and β-actin (rabbit polyclonal antibody 1:2000) (Cell Signaling Technology, Beverly, MA, USA) and its HRP-labeled secondary antibodies [18], followed by detection with an enhanced chemiluminescence reagent. The chemiluminescence of expressed bands was examined to confirm the linear range of the chemiluminescence signals, and the quantifications were performed using the densitometry tool in ImageJ software v.1.8.

### 2.15. Statistical Analysis

Data were expressed as mean  ±  SEM and the differences between variables were analyzed using one-way analysis of variance (ANOVA) with multiple comparisons of groups. The repeated measure test was used to analyze clinical scores and body weight over the time course of the whole investigation. Significant differences were considered when *p* < 0.05. All data were analyzed using Excel data sheet-10 and GraphPad Prism software v.6.01.

## 3. Results

### 3.1. Effect of Immunomodulation and Biocompatibility of BA on HT-29 Colon Cell Lines

**Figure 1:**
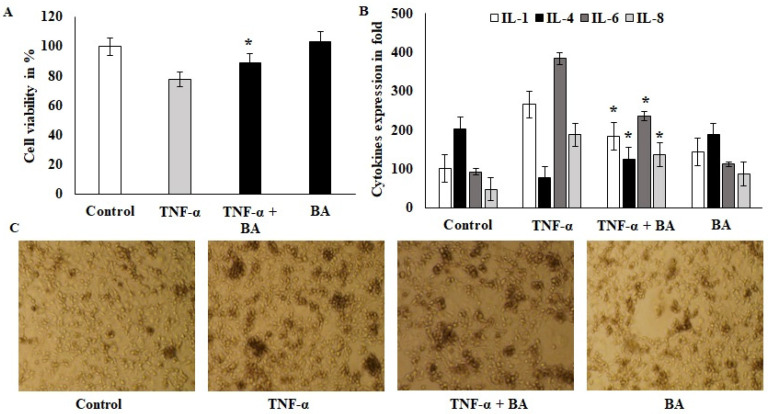
Effect of probiotics on colon cells: (**A**) BA 10^8^ microbial loads treated with ht29 colon cells and incubated for 2 h; after incubation, the 6-well plates were aspirated with ampicillin 20 U/mL, and the treated cells were quantified for cell viability using 4% trypan blue. (**B**) TNF-α-induced HT29 cells were treated with BA for 12 h and the cell-free supernatant was collected and used for cytokine IL1, IL4, IL6 and IL8 estimation and the values were expressed as pg/mL. All of the data were collected from three individual experiments and pooled and expressed as mean ± SD (*p* < 0.05). (**C**) Phase-contrast images of TNF-α-induced HT29 cells that were treated with BA for 2 h; the magnification in the microscopic image was 200×. * *p* < 0.05 represents significance compared to the TNFα vs. TNFα + BA group.

A viability exclusion test was used to assess the cytotoxic effects of BA on HT-29 cell lines. Confluence cells were challenged with 10 ng/mL of TNF-α for 1 h, after which the co-culture with BA showed protective effects and the cell viability was significantly improved compared to the TNF-α alone treated group (Figure 1A). BA alone treated cells did not show toxicity and indicated that no toxic components or endotoxins existed in *BA*. The immunomodulatory effect of BA was tested on this same model with an increased incubation period and showed downregulated pro-inflammatory cytokines such as IL-1 and IL-6. The values of IL-1 and IL-6 were 273 pg/mL (TNF-α-induced) to 197 pg/mL (*BA*–treated) and 382 to 211 pg/mL, respectively. The anti-inflammatory marker IL-4 was upregulated by BA treatment compared to TNF-α-induced cells (Figure 1B). Interestingly, IL8, a powerful neutrophil chemoattractant, was increased in TNF-α-induced cells, whereas BA treatment reduced the IL8 expression from 201 to 98 pg/mL concentration (Figure 1B). Inflamed HT-29- and BA-treated cells were observed at 200× objective view under a phase contrast inverted microscope (Figure 1C). The granulation and cellular degradation were observed in TNF-α induction and they were significantly reduced in BA-treated cells. The cell sphere formation and clumping indicate the inflamed morphology and it was reduced in BA-treated cells. These in vitro results showed that BA improves the cell viability and anti-inflammatory characteristics via cell structural regulations (Figure 1C).

As can be seen in Table 2 the nutritional content of BEY was increased and total fat, protein, and total carbohydrates were increased relative to the commercial product (Table 2). These results were according to in vivo immunopathological studies.

### 3.2. BEY Alleviated TNBS-Induced Colitis in C57Bl6j Mice

**Figure 2:**
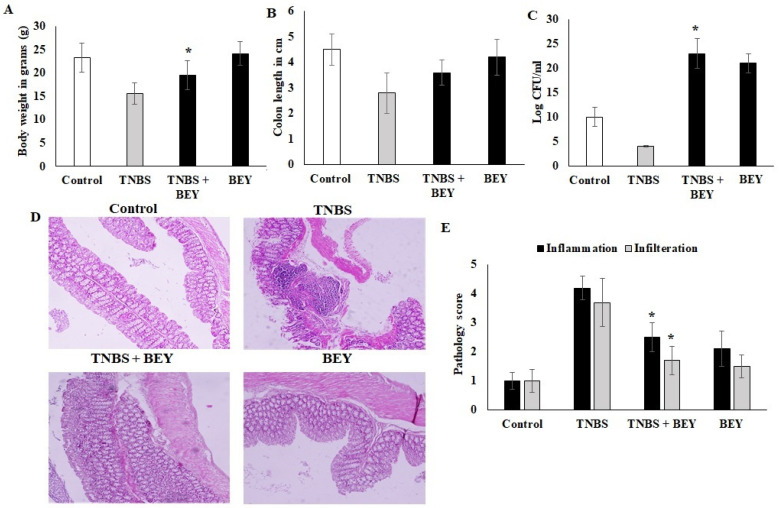
BEY alleviated TNBS-induced colitis in C57Bl6j mice. TNBS induction was made at 0 and 4 days of induced mice; BA 10^8^/mL was administered as oral gavage at alternative days from 0 to 14. (**A**) the body weight of BA-treated TNBS-induced colitis mice and the values were expressed in g. The BA alone group was analyzed in parallel with the TNBS-induced group. (**B**) Colon lengths of TNBS-induced colitis mice and BEY-treated mice were evaluated and values were expressed in cm. (**C**) The fecal microbial load was quantified in TNBS-induced colitis mice and BA-treated mice, and values were expressed as log CFU/mL. (**D**) Microscopic examination of distal colon of TNBS-induced colitis was carried out, and the pathological changes were observed using the H&E staining method. The pathological score was evaluated in TNBS-induced colitis mice and BA-treated mice. The cellular infiltration and inflammation were observed, and the magnification in the microscopic image was 200×. (**E**) The pathological score distal colon was evaluated in TNBS-induced colitis mice and *Bacillus*-treated mice. Cellular infiltration and inflammation were observed. All of the data were collected from three individual experiments and pooled and expressed as mean ± SD (*p* < 0.05). * *p* < 0.05 represents significance compared to the TNBS vs. TNBS + BEY group.

The study demonstrated the potential therapeutic effect of BEY on mice with TNBS-induced colitis. Mice induced by TNBS displayed a severe illness with fecal heme leakage, and consequently, they sustained weight loss and bloody diarrhea (Figure 2A,B). The colon length and fecal microbial load showed the recovery of the disease. BEY ameliorated the weight loss (~30%), improved colon length (~30%), and ameliorated disease severity with a survival rate of 85.7%. The total fecal microbial load was increased in the BEY-treated group compared to the TNBS alone mice group (Figure 2C). The average colon length of the BEY-treated mice was longer than that of the TNBS group (Figure 2B,C). Histological evaluation of the distal colon of the BEY-treated mice showed neutrophil infiltration and inflamed dysplasia at the mucus of villi (Figure 2D,E); the distal colon was inflamed and heme leakage was significantly observed in TNBS-induced mice groups (Figure 2E). The neutrophil infiltration was correlated with the following evaluation of colon MPO levels.

### 3.3. Effect of BEY on Inflammatory Markers in TNBS-Induced Colitis Mice

**Figure 3:**
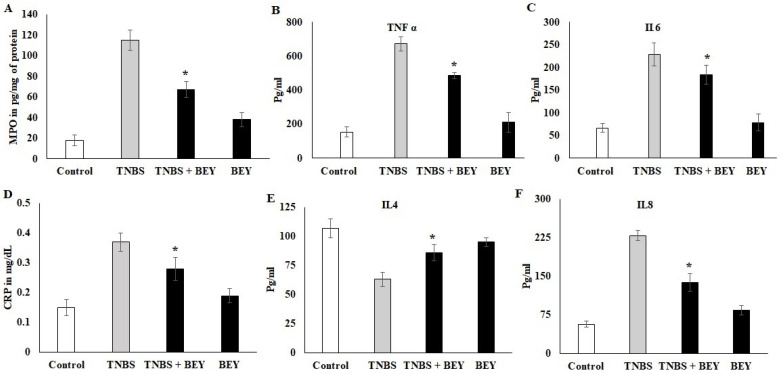
Effect of BEY on inflammatory markers in TNBS-induced colitis mice. The myeloperoxidase-infiltration marker was quantified in TNBS-induced colitis mice and BEY-treated mice after 14 days of the experiment. The values are expressed in pg/mg protein. (**A**) the neutrophil infiltration marker MPO was quantified; (**B**,**C**) TNF-α and IL-6 (pro-inflammatory markers) were quantified in TNBS-induced colitis mice and BEY-treated mice after 14 days of the experiment. The values are expressed in pg/mL. (**D**) The lymphocytes degradation marker (C-reactive protein) was quantified in TNBS-induced colitis mice and BEY-treated mice after 14 days of the experiment. The values are expressed in mg/dL. (**E**,**F**) IL4 and IL8 (anti-inflammatory and macrophage markers, respectively) were quantified in TNBS-induced colitis mice and BEY-treated mice after 14 days of the experiment. The values are expressed in pg/mL. All data were collected from three individual experiments and pooled and expressed as mean ± SD (*p* < 0.05). * *p* < 0.05 represents significance compared to the TNBS vs. TNBS + BEY group.

The activity of MPO was determined in the colonic homogenate of the TNBS-induced and BEY-treated mice groups. The results displayed considerable changes in the MPO activities between the treated and control groups in TNBS models (Figure 3A). The MPO activity was significantly increased in the TNBS colitis group (117.45 ± 8.5091 pg/mL) compared to the non-colitis (17.1 ± 0.9483 pg/mg of tissue) and BEY-treated groups (61.4 ± 8.087 pg/mg of tissue). In comparison to the TNBS colitis group, a significant reduction in MPO levels was observed in the BEY-treated group (*p* < 0.01). Overall, the BEY-treated TNBS-induced group showed the most notable reduction (*p* < 0.05) in MPO activity among all of the treatment groups. A comparative evaluation of cytokines (TNF-α and IL-6) in the colonic homogenate of different mice groups in a TNBS-induced colitis model is illustrated in Figure 3B,C. TNBS colitis mice showed elevated levels of TNF-α and treatment groups showed a remarkable decrease in their level. Significant variation was observed in IL-6 expression levels compared to the TNBS-induced and BEY-treated mice groups (*p* > 0.062).

CRP levels showed a negative expression by BEY treatment compared to the TNBS-induced group (>0.042). It was significant in both induced and non-induced mice groups (Figure 3D). In the case of the anti-inflammatory cytokine IL-4, the levels were significantly (*p* < 0.05) low in the colonic homogenate of the TNBS colitis mice group (49.1 ± 3.20 pg/mL) compared to the non-colitis group (108.9 ± 8.2 pg/mL). The administration of BEY showed (72 ± 4.8) a positive effect and substantially enhanced the secretion of IL-4 levels (*p* < 0.001). Additionally, the macrophagic secretion marker IL-8 was decreased compared to the TNBS mice group from 243 to 139 pg/mL (*p* < 0.05). It is clear that BEY exhibited a stronger modulatory effect on the secretion of inflammatory cytokines in the intestines of mice.

### 3.4. Effect of BEY on Intestinal Barrier Function of TNBS-Induced Colitis Mice

**Figure 4:**
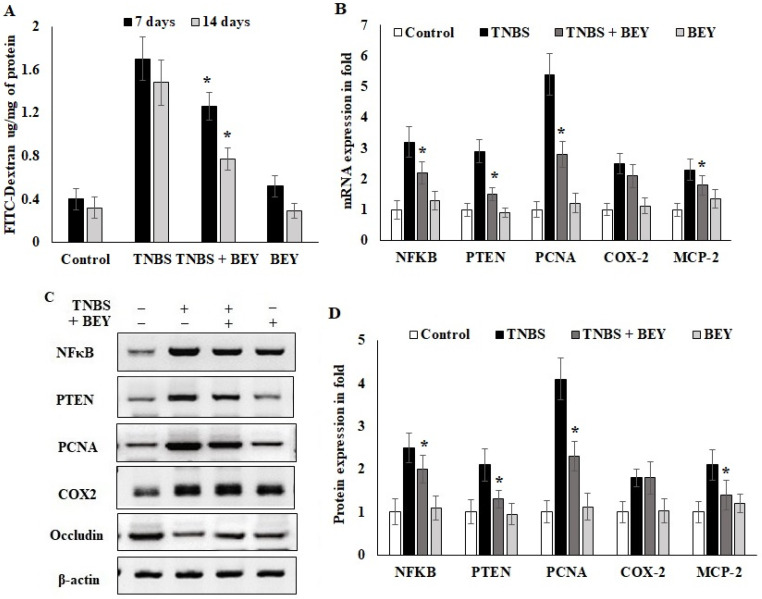
Effect of *Bacillus* on TNBS-induced colitis. *Bacillus* enhanced the intestinal barrier function of colitis mice by regulating inflammatory markers. (**A**) Detection of FITC-dextran in serum of mice by fluorometric ELISA. (**B**) qPCR of NFκB, PTEN, PCNA, COX-2 and occludin mRNA in colonic tissues on the 14th day of TNBS-induced mice (all values were normalized to the TNBS-induced group). (**C**,**D**) Immunoblot and densitometric analysis of NFκB, PTEN, PCNA, COX-2 and occludin protein expression normalized with actin internal control. Data are representative of at least three independent experiments. Data are shown as mean ± SD of the mean (*n* = 3). * *p* < 0.05 represents significance compared to the TNBS vs. TNBS + BEY group.

Epithelial cell barrier function loss is an intestinal disorder, including IBD. We studied barrier function in water-drinking mice and TNBS-treated mice using a FITC-labeled dextran method, as described in the methodology section. Mice were administered FITC-dextran by gavage, and fluorescence was quantified in the serum at 4 h after the administration of FITC-dextran. As shown in Figure 4A, water-drinking mice showed a FITC-dextran of 0.42 μg/mg of FITC/mg protein. In comparison, there was a 3.5-fold increase in the FITC-dextran levels in the TNBS mice group on day 7 (1.75 mg of FITC) and day 14 (1.42 μg/mg protein) compared to the control mice. The results of BEY treatment showed 1.24 and 0.74 μg/mg protein for day 7 and day 14 samples, respectively. These results suggest that improved barrier function was observed in BEY-treated mice.

In order to understand the inflammatory marker expression in the histologically affected distal colon compared to a relatively non-affected control colon, real-time PCR was performed to evaluate the expression level of inflammatory markers in the TNBS and BEY-treated colitis mice groups. The mRNA expressions of NFκB, PTEN and PCNA significantly increased in the TNBS-induced mice groups, whereas BEY treatment reduced the significant level of all markers except COX-2 compared to the TNBS-induced group (*p* > 0.04) (Figure 4B). COX2 expression was comparatively less significant than other tested markers (*p* > 0.0625). Protein expression also reflects the mRNA expression profile, but COX2 showed insignificant changes between the TNBS induction and BEY-treated groups (Figure 4C,D). Interestingly, the integrity marker occludin was downregulated by TNBS induction and significant recovery of intestinal permeability was reflected in upregulation in the BEY treatment (Figure 4D). In overview of the results, the BEY-induced protective effects on epithelial barrier integrity are mediated by the PTEN/PCNA pathway; highly significant down-regulation was measured, and the PCNA load in colon epithelial tissues was further confirmed using immune histopathological examinations.

### 3.5. Immunohistochemical Expression of PCNA in Colon Tissues of TNBS-Induced Mice

**Figure 5:**
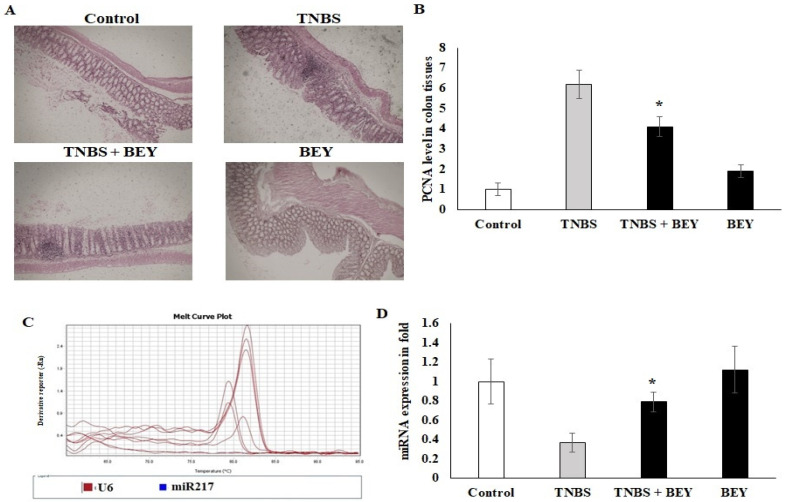
Immunohistochemical expression of PCNA in colon tissues of control and experimental groups of mice. PCNA protein expression is illustrated as brown staining. (**A**) Microscopic imaging of colon tissue with scale bar: 100 µm. (**B**) The quantitative data expressing the high-grade dysplasia marker PCNA protein level illustrate that there was significant downregulation in the TNBS with *Bacillus* and *Bacillus* alone groups compared with the TNBS alone disease group. (**C**,**D**) The epigenetic marker miR-217 was quantified using real-time PCR. Data are shown as mean ± standard error of the mean (*n* = 3). * *p* < 0.05 represents significance compared to the TNBS vs. TNBS + BEY group.

The influence of TNBS on the colon tissue of mice was observed with respect to the marker of PCNA, which was detected by immunohistochemistry. Figure 5A shows immunohistochemical staining of the mice colon sections with PCNA-positive cells located in the periphery of the upper epithelial tissue and in the lamina propria. The values expressed as relative optical density (ROD) of the section showed a significantly reduced cellular proliferation (*p* < 0.01) in the BEY alone and BEY with TNBS-induced mice groups. These results confirm that BEY attenuated the negative effects of TNBS on cell proliferation in the colitis.

The PCNA expression was further confirmed by microRNA expression of miR217. TNBS induction significantly downregulated the expression level of miR217 (0.32 ± 0.08), whereas its expression was substantially upregulated by BEY-treatment (Figure 5C,D). These results demonstrate that BEY treatment affects the colitis via the PCNA/miR217 axis.

## 4. Discussion

Recently, probiotic fermented camel milk gained importance due to its contributions to immunity, gastrointestinal health and metabolic homeostasis. It has been seen that it has a prophylactic and ameliorative effect on inflammatory disorders in humans as well as in animals. Additionally, it increases the beneficial bacteria in the gastrointestinal tract (GIT), such as *Bifidobacterium* and *Lactobacillus* [19,20], and decreases the harmful ones, e.g., *Escherichia* and *Shigella* [4]. It has been previously reported that camel milk ameliorated DSS-induced colitis via regulating the gut microbiota and intestinal barrier and reducing proinflammatory factors [4]. However, little is known about the effects of probiotic-fermented camel milk on TNBS-induced colitis. Therefore, this study was conducted to bridge this gap. The probiotic bacterium, BA, which was isolated from soil samples by Hairul et al. [10], displayed an initial beneficial impact on the DSS-induced colitis mice model. For further investigation, BA was used in this study to ferment camel’s milk and its improving effects on a TNBS-induced colitis model were assessed.

The safety assessment of BA on HT-29 human colon cell lines was demonstrated by no loss of cell viability and prevention of the cytotoxicity effects induced via TNF-α. Similar results were obtained on *L. plantarum* and *L. acidophilus* [21] and *B. coagulans* and *B. subtilis* [22]. Our observations demonstrate that BA does not possess any endotoxins and hence is a biocompatible bacterium to the colon cells, for the subsequent immunomodulation investigations on TNBS-induced colitis model.

Host–probiotic interactions improve gut immune homeostasis and regulate inflammatory markers [21]. The reduction in IL-1, IL-6 and IL-8 levels in TNF-α-induced cells can be explained by the ability of BA to suppress anti-inflammatory mediators, as further confirmed in an in vivo model. Comparable findings were obtained in many in vitro studies. *L. acidophilus*, *B. coagulans* and *B. subtilis* exhibited potential anti-inflammatory effects on Caco2, dendritic and PBM cells [22,23,24]. These results confirm that gut laminal and epithelial cells of mucous play a major role in probiotic interactions via secreting inflammatory mediators such as chemoattractant proteins, interleukins and infiltration signals.

TNBS-induced colitis mice are useful models to study pathogenesis, histopathology and treatment approaches of IBD, as they represent the human IBD in the preclinical model [25]. In the current study, many severe clinical, histopathologic and immunohistopathology features of colitis were reported in the TNBS-treated mice models compared to untreated ones, including bloody stool, diarrhea, weight loss leading to a high value of DAI and shortening of colon length. Additionally, the histopathologic analysis results of the colon tissues in TNBS-included mice included erosion of epithelial cells, damage in goblet cells, crypts, apoptosis, inflammation, neutrophil infiltration and inflamed dysplasia at the mucus of villi, as well as high pathology inflammation scores. BEY treatment improved colon health by inhibiting the progression of colitis, which has been seen in previous studies consistently [4,26,27]. Cui et al. [26] showed that camel milk regulated T-cell proliferation to alleviate colitis in a DSS-induced mice model. Similarly, Kangwan et al. [27] reported that *L. pentosus*, which was obtained from fermented tea leaves, improved colon abnormality [27]. Treatment of probiotics significantly protected the colon from being inflamed by mitigating histological and clinical damage traits, enhancing the intestinal barrier integrity, and attenuating inflammation symptoms induced by TNBS. These results suggest that BEY possesses substantial ameliorative effects in TNBS-induced mice models.

Inflammation activity is mediated by inflammatory cytokines and their amount is proportional to the inflammation status [28]. The dysfunction of the mucosal barrier in colitis results in dysregulation in immune responses leading to damaged and inflamed tissues.

Oxidative stress and colon inflammation are closely related to IBD, and TNBS initiates the lipid peroxidases (MDA and NO) [29]. In this study, BEY treatment enhanced antioxidant activities as indicated by a remarkable reduction in MPO activity in TNBS-induced colitis. These findings are consistent with those reported in previous studies [30,31]. Treatment with BEY significantly suppressed the proinflammatory secretion levels of TNF-α, IL-6, MPO and CRP, compared to the control, indicating the anti-inflammatory and immunomodulatory efficacy of BEY. Comparable results were obtained in previous studies [32,33]. Furthermore, the anti-inflammatory cytokine IL4 was upregulated relative to the TNBS-induced group. The chemoattractant cytokine IL8 showed a distinct expression pattern and controlled neutrophil infiltration in inflamed tissues of the BEY- treated mice. These results confirm the histological examination of infiltration levels. Therefore, BEY possesses an efficient anti-inflammatory and immunomodulatory probiotic.

The transcription factor NFκB has a key role in immune and inflammatory responses. The BEY treatment from our study downregulated the TNBS-induced mice colitis. Similarly, the syringic and ascorbic acids inhibited the NFκB in N-Nitrosodimethylamine induced pulmonary inflammation [34]. Conversely, lower levels of PTEN were observed in both TNBS and N-Nitrosodimethylamine treatments, while they were increased after BEY and syringic and ascorbic acids intervention [34]. PCNA, the conserved protein responsible for epithelial proliferation through the Wnt/β–catenin pathway, showed increased levels after BEY administration to the TNBS-induced colitis model comparable to the probiotic strain *L. reuteri* 22 observed in young chickens [35]. Cox-2 along with NFκB induced a higher inflammatory response in TNBS-induced mice colitis; however, BEY treatment showed a reduction in their level, which was also seen with the probiotic *L. rhamnosus* along with Celecoxib [36]. The occludin and inflammatory markers indicate intestinal barrier integrity and stability of host health. TNBS has a devastating effect on intestinal mucosa and disrupts its epithelial barrier. Once there is a disruption, it creates permeability to immune responses and inflammation [37]. Therefore, the higher immune response results in higher inflammatory cytokines secretion, leading to impaired intestinal barrier function associated with the occludin and claudin proteins [38,39]. Hence, these proteins are responsible for the integrity of the membrane and its function in tight junctions [40,41]. Our results showed that BEY treatment has significant roles in maintaining the intestinal barrier integrity and preventing colitis, as evidenced by the increased levels of occludin and negative regulation of inflammatory markers in colitis mice.

The gut microbiota has a key role in maintaining GIT health. One potential explanation of the ameliorative effects of BEY on TNBS-induced colitis is due to modulating the diversity and abundance of the GIT microbiota, which was not studied here. Unlike TNBS or DSS, camel milk provides an environment to sustain beneficial bacteria [4]. The microbiota in camel’s milk synthesizes short chain fatty acids (SCFA), which are essential to intestinal health by inhibiting the inflammatory cytokines and preserving the intestinal barrier [42].

The inflamed colonic cells expressed significant levels of cell proliferative and inflammatory mediators including PCNA. PCNA targeting potential miRNA (microRNA217) was identified using a target scan [43], which showed reciprocal regulation to the functional expressions of PCNA. BEY repressed TNBS-induced PCNA activation and upregulated miR217 in colonic tissue. Congruent results were observed by Zhang et al. [44], who found that miR26 attenuated colitis by targeting the intestinal inflammatory pathways [44]. This finding indicates that negative regulation of PCNA was achieved via the interaction of BEY with the epigenetic factor microRNA217.

## 5. Conclusions

In the present study, BA has shown immunomodulatory effects on TNF-α induced HT29 colon cells. BEY ameliorated the colitis disease index and improved the body weight and colon length of TNBS-induced colitis mice. Pro-inflammatory cytokines, inflammatory progress mRNA and protein markers were significantly downregulated by BEY treatment accompanied by mucosal barrier integrity. Interestingly, significant suppression of PCNA was observed in BEY treatment through the epigenetic microRNA217 interaction. In conclusion, BEY clearly alleviated colitis symptoms and in the future could be used to formulate a probiotic-based diet for host gut health and control inflammatory bowel syndrome in mammals. The overall findings reported in this study support the substantial ameliorative effects in TNBS-induced mice model and could serve as a novel strategy for the protection of colon inflammation.

## Figures and Tables

**Table 1:** Primer details of the mRNA target.

Primer Name	Forward Primer	Reverse Primer	PCR Product Size in bp
NFκB	CATGAAGAGAAGACACTGACCATGGAAA	TGGATAGAGGCTAAGTGT AGACACG	245
PTEN	ATGACAGCCATCATCAAAGAGATCGTTAG	GGGTCAGACTTTTGTAATTTGTGAATGCTG	148
PCNA	TGC TCT GAG GTA CCT GAA CT	TGC TTC CTC ATC TTC AAT CT	189
COX2	CAA TTC CCG GAC GTC TAA ACC	CTA GGA CGA TGG GCA TGA AAC	114
OCCLUDIN	ACG TCC GAC CCA TGC TCT CT	AAG TCA TCC GCA GGG GAG GT	147
GAPDH	TGGCCTACATGGCCT CCA	TCCCTAGGCCCCTCCTGTTAT	177

**Table 2:** Nutritional content of BEY (~100 mL) in comparison with commercial yogurt.

Nutritional Content	BEY—Values in Grams (g)	Commercial Yogurt—Values in (g)
Total fat	3.4 ± 0.2	3.22 ± 0.13
Trans fat	0.0	0.0
Cholesterol	0.049 ± 0.01	0.051 ± 0.007
Sodium	0.04 ± 0.01	0.035 ± 0.1
Total carbohydrates	4.5 ± 0.31	4.1 ± 0.24
Protein	3.2 ± 0.28	2.92 ± 0.2

## Data Availability

Available from corresponding author upon request.
